# Ultrasound guided injection of dexamethasone versus placebo for treatment of plantar fasciitis: protocol for a randomised controlled trial

**DOI:** 10.1186/1757-1146-3-15

**Published:** 2010-07-16

**Authors:** Andrew M McMillan, Karl B Landorf, Mark F Gilheany, Adam R Bird, Adam D Morrow, Hylton B Menz

**Affiliations:** 1 Department of Podiatry, Faculty of Health Sciences, La Trobe University, Victoria, Australia; 2 Musculoskeletal Research Centre, Faculty of Health Sciences, La Trobe University, Victoria, Australia

## Abstract

**Background:**

Plantar fasciitis is the most commonly reported cause of chronic pain beneath the heel. Management of this condition commonly involves the use of corticosteroid injection in cases where less invasive treatments have failed. However, despite widespread use, only two randomised trials have tested the effect of this treatment in comparison to placebo. These trials currently offer the best available evidence by which to guide clinical practice, though both were limited by methodological issues such as insufficient statistical power. Therefore, the aim of this randomised trial is to compare the effect of ultrasound-guided corticosteroid injection versus placebo for treatment of plantar fasciitis.

**Methods:**

The trial will be conducted at the La Trobe University Podiatry Clinic and will recruit 80 community-dwelling participants. Diagnostic ultrasound will be used to diagnose plantar fasciitis and participants will be required to meet a range of selection criteria. Participants will be randomly allocated to one of two treatment arms: (i) ultrasound-guided injection of the plantar fascia with 1 mL of 4 mg/mL dexamethasone sodium phosphate (experimental group), or (ii) ultrasound-guided injection of the plantar fascia with 1 mL normal saline (control group). Blinding will be applied to participants and the investigator performing procedures, measuring outcomes and analysing data. Primary outcomes will be pain measured by the Foot Health Status Questionnaire and plantar fascia thickness measured by ultrasound at 4, 8 and 12 weeks. All data analyses will be conducted on an intention-to-treat basis.

**Conclusion:**

This will be a randomised trial investigating the effect of dexamethasone injection on pre-specified treatment outcomes in people with plantar fasciitis. Within the parameters of this protocol, the trial findings will be used to make evidence-based recommendations regarding the use of corticosteroid injection for treatment of this condition.

**Trial Registration:**

Australian New Zealand Clinical Trials Registry. ACTRN12610000239066.

## Background

Plantar fasciitis is the most commonly reported cause of chronic pain beneath the heel [1,2]. The condition is characterised by pain at the calcaneal origin of the plantar fascia, made worse by weight-bearing after prolonged periods of rest [1]. The epidemiology of plantar fasciitis in the general population is currently uncertain. An Australian population-based study involving 3,206 randomly selected participants has reported a heel pain prevalence of 3.6% [3]. A North American study of adults aged over 65 years found that 7% had tenderness beneath the heel [4]. It has also been estimated that 1 million physician visits per year in the United States are for the diagnosis and treatment of plantar fasciitis [5]. In addition, the disorder is estimated to account for approximately 8% of all running-related injuries [6,7].

The underlying pathology of plantar fasciitis is poorly understood, though the majority of histological studies report a predominance of degenerative changes at the plantar fascia enthesis. The most common pathological features are deterioration of collagen fibres, increased secretion of ground substance proteins, focal areas of fibroblast proliferation and increased vascularity [8-11]. The presence of biochemical markers of inflammation (e.g. cytokines and prostaglandins) have not been well investigated, however, several studies report non-specific evidence of local inflammatory change [11-13]. As described in relation to tendinopathy [14], it is feasible that plantar fasciitis is a disorder that proceeds through a spectrum of underlying processes. However, evidence for histological change over time is currently lacking, as current studies have only examined specimens obtained from patients undergoing surgery for long-standing symptoms. Therefore, the underlying pathology occurring early in the development of plantar fasciitis is currently unknown.

Plantar fasciitis is commonly described in the literature as a self-limiting condition [1,2]. This view is supported by the findings of a systematic review, in which plantar heel pain was found to resolve over time regardless of treatment type (including placebo) [15]. Nonetheless, plantar fasciitis can be a very painful and disabling condition prior to resolution of symptoms, causing a negative impact on health-related quality of life [16].

Many interventions are used for the management of plantar fasciitis [17] and corticosteroid injection is a common choice among clinicians. Surveys of American podiatrists [18] and orthopaedic surgeons [19] have reported that approximately 75% of respondents used and/or recommended this intervention. In addition, a systematic review found that corticosteroid injection is the second most frequently described treatment for plantar fasciitis in the medical literature [20].

Despite the widespread use of corticosteroid injection for plantar fasciitis, only two randomised controlled trials have tested the effect of this treatment in comparison to placebo injection [21,22]. By using placebo solution as a comparator, these trials were able to control for potential treatment benefits not due to the pharmacological action of corticosteroids. One trial compared the effect of 25 mg prednisolone mixed with lignocaine versus lignocaine alone (placebo), and found a significant difference in pain reduction favouring corticosteroid one month after treatment [22]. No significant differences between groups were detected in this trial at either three or six months after treatment. However, a large proportion of participants were lost to follow-up, so the authors were unable to make conclusions regarding corticosteroid efficacy in the longer term. An earlier trial compared the effect of 25 mg hydrocortisone versus normal saline (placebo), and found no significant difference in pain reduction between groups two months after treatment [21]. However, this trial had a very small sample size (19 participants) and was therefore statistically underpowered to detect clinically worthwhile differences.

The use of ultrasound in clinical practice has become increasingly popular due to decreased equipment costs, and the ability to perform invasive procedures with better targeting of anatomical structures [23]. Other advantages include the production of high resolution images without exposure to ionizing radiation, and the ability to assess tissues with real-time dynamics [23]. Furthermore, in the treatment of plantar fasciitis, corticosteroid injection performed with ultrasound guidance has been shown to produce longer lasting pain relief than injection guided by palpation [24].

The findings of existing clinical trials provide some support for the use of corticosteroid injection in the short-term management of plantar fasciitis [1,15]. However, a recent systematic review concluded that the effectiveness of this treatment has not been sufficiently established [17], indicating that further research is required. Therefore, the aim of this trial is to investigate the effectiveness of ultrasound-guided corticosteroid injection for treatment of plantar fasciitis over a 12 week period.

## Methods

The trial has been registered on the Australian New Zealand Clinical Trials Registry (ACTRN12610000239066). Recruitment of participants commenced on 3^rd ^June 2010 and is expected to continue until March 2011.

### Ethical approval

The La Trobe University Human Ethics Committee has approved the trial (Application Number: 09-062) and all participants will provide written informed consent prior to enrolment. Ethical standards will adhere to the National Health and Medical Research Council (NHMRC) National Statement [25] and the World Medical Association's Declaration of Helsinki [26]. Publications associated with the trial will be formatted according to the CONSORT Statement [27].

### Setting and eligibility criteria

The trial will be conducted at the La Trobe University Podiatry Clinic and will recruit local community-dwelling participants by multiple newspaper advertisements. Participants will be required to have a clinical history of pain beneath the heel for at least eight weeks prior to enrolment, and to report a minimum heel pain magnitude of 20 mm on a 100 mm visual analogue pain scale. On clinical examination, participants will also be required to report sensitivity to palpation of the medial calcaneal tubercle and/or the proximal plantar fascia. To confirm the diagnosis of plantar fasciitis, the dorso-plantar thickness of the plantar fascia will be measured by ultrasound at a standard location where the fascia crosses the anterior aspect of the inferior calcaneal border. Participants will be required to have a plantar fascia thickness value of 4.0 mm or greater [28]. Finally, participants will be required to attend the second visit (during which a tibial nerve block and heel injection will be given) with a friend or family member who is able to provide transportation from the clinic.

Applicants will be excluded from the study if they have received a corticosteroid injection for plantar fasciitis within the previous six months, or if they have any of the following: a known hypersensitivity to lignocaine hydrochloride or corticosteroids, current skin or soft tissue infection near the injection site, posterior heel pain, current pregnancy, systemic inflammatory disease, diabetes mellitus, previous local surgery, or a history of local trauma. Applicants will also be excluded if they are unable to walk household distances without the use of an aid, or if they have commenced any treatment regimen for plantar fasciitis within four weeks prior to enrolment.

**Figure 1:**
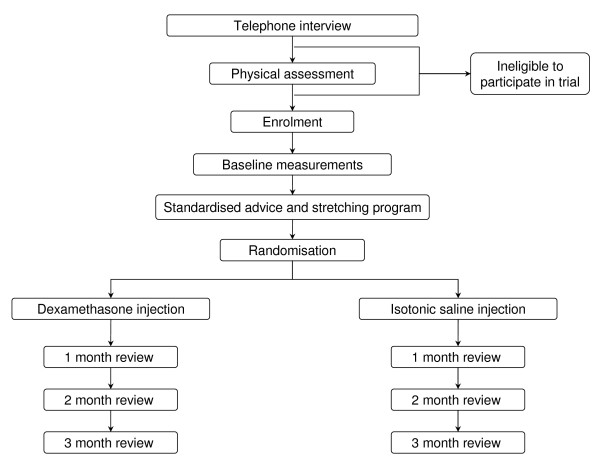
**Trial profile**.

Screening of applicants according to these criteria will occur by a preliminary telephone interview, followed by clinical examination at the initial visit. After a detailed explanation of the study protocol, eligible applicants will be invited to participate in the trial. However, prior to enrolment, all applicants will be assessed for competence to give consent by use of the Evaluation to Sign Consent (ESC) tool [29]. The ESC is specifically designed for use in clinical research and has been shown to have good inter-rater reliability (Pearson *r *= 0.81) [30]. A range of descriptive characteristics will also be collected at the initial visit after enrolment (Table 1). Participants will then be scheduled to attend a second appointment (approximately one week later) where baseline measurements will be taken and the trial intervention performed (Figure 1).

**Table 1:** Descriptive characteristics

Age
Sex
Height
Weight
Body Mass Index (BMI)
Duration of symptoms
Previous and current treatments

### Interventions

Participants will be randomly allocated to one of two treatment arms: (i) ultrasound-guided injection of the plantar fascia with corticosteroid (experimental group), or (ii) ultrasound-guided injection of the plantar fascia with normal saline (control group). Prior to plantar fascia injection, participants in both groups will be given an ultrasound-guided posterior tibial nerve block with 2% lignocaine hydrochloride. All participants will be positioned prone on a treatment table with their knees extended. Corticosteroid injection will be performed with a 25 gauge (38 mm) needle and a 1 mL Luer-lock syringe containing 1 mL of 4 mg/mL dexamethasone sodium phosphate. Saline injection will be performed with a 25 gauge (38 mm) needle and a 1 mL Luer-lock syringe containing 1 mL normal saline (0.9% sodium chloride). For both injections, the needle will be inserted through the medial heel perpendicular to the long axis of the ultrasound transducer, and will be advanced under continuous guidance into the proximal plantar fascia [31]. Infiltration will occur near the calcaneal enthesis in the region of maximal fascia thickening. An aseptic technique will be used for all injections to minimise infection risk, including use of sterile gloves, sterile transducer covers and sterile transmission gel. Participants with bilateral plantar fasciitis will have both feet treated (with their allocated intervention) during a single appointment. Following treatment, participants will be advised to avoid all running and other high impact activities for at least 2 weeks.

Throughout the initial 8 weeks of enrolment, all participants will be required to complete a daily stretching program shown to decrease pain associated with plantar fasciitis [32]. The stretching technique requires participants to cross their affected leg over the contralateral knee in a seated position, then pull back on the toes until they feel a stretch in the arch of their foot. Participants will be instructed to repeat the stretch 10 times, with each stretch lasting for 10 seconds. All participants will be asked to complete the stretching program three times per day and to record their stretching frequency in a diary.

### Randomisation, treatment allocation and blinding

Treatment allocation will be performed according to a computer-generated randomised number sequence. Allocation will be concealed in a password protected computer file accessible by an investigator not involved in measuring outcomes (ADM). This investigator will also prepare the syringe prior to heel injection, thereby ensuring the investigator giving injections, measuring outcomes and analysing data (AMM) is blinded throughout the trial duration. As both treatment solutions (i.e. dexamethasone sodium phosphate and normal saline) appear in the syringe as clear liquids free from visible particulate matter, the syringe contents will not require masking. This protocol will also ensure that trial participants are blinded to their treatment allocation.

### Primary outcomes

**Figure 2:**
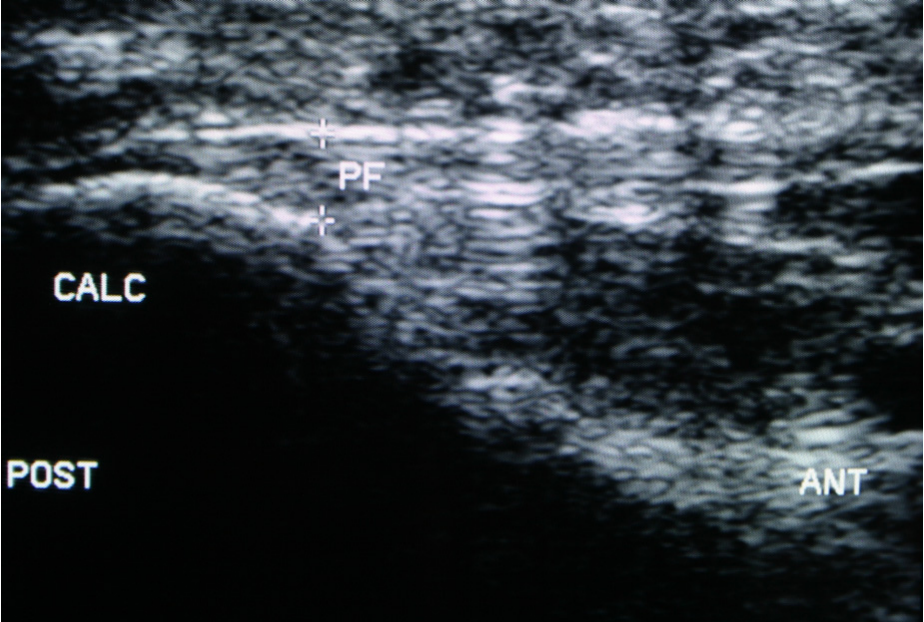
**Plantar fascia thickness: example of measurement location**.

Primary outcomes will be pain and plantar fascia thickness at 4, 8 and 12 weeks. Pain will be measured by the foot pain domain of the Foot Health Status Questionnaire (FHSQ), which has been shown to have a high degree of internal consistency (Cronbach's α = 0.88) and test-retest reliability (intra-class correlation coefficient = 0.86) [33]. Participants treated for bilateral plantar fasciitis will be asked to describe symptoms without specific reference to an individual foot (i.e. bilateral pain will be evaluated as one independent sample). Plantar fascia thickness will be measured by ultrasound at a standard location where the fascia crosses the anterior aspect of the inferior calcaneal border (Figure 2). This measurement technique has been shown to have good intra-rater reliability, with the 95% limits of agreement ranging from -0.7 mm to 0.5 mm [34]. Participants treated for bilateral plantar fasciitis will have thickness measurements taken for each individual foot. However, for bilateral cases, the mean change in plantar fascia thickness for the two feet will be calculated at each follow-up in order to evaluate data as one independent sample. Plantar fascia measurements and ultrasound guided injections will be performed with a variable frequency (5-10 MHz) linear array transducer (Acuson Aspen, Siemens Medical Solutions, Pennsylvania, USA).

### Secondary outcomes

Secondary outcomes will be function, use of oral analgesic medication, and 'first-step' pain at 4, 8 and 12 weeks. Function will be measured by the foot function domain of the FHSQ, which has been shown to have a high degree of internal consistency (Cronbach's α = 0.85) and test-retest reliability (intra-class correlation coefficient = 0.92) [33]. Participants treated for bilateral plantar fasciitis will be asked to describe foot function without specific reference to an individual foot (i.e. bilateral function will be evaluated as one independent sample). Use of oral analgesic medication (including paracetamol, aspirin, opioids and non-steroidal anti-inflammatory drugs) will be recorded at each visit. 'First-step' pain, experienced when initially rising from bed in the morning, will be measured on a 100 mm visual analogue scale. Participants treated for bilateral plantar fasciitis will be asked to indicate the magnitude of symptoms without specific reference to an individual foot (i.e. bilateral pain will be evaluated as one independent sample).

### Sample size

Prospective sample size calculation indicates a sample of 40 participants per group (i.e. 80 total) would provide 80% power to detect a minimal important difference of 13 points [35] on the pain domain of the FHSQ (SD = 20, alpha = 0.05, 5% loss to follow-up). The extra precision provided by covariate analysis was conservatively ignored when performing this calculation.

### Adverse events

Complications and adverse events associated with the intervention (e.g. infection, nerve injury or plantar fascia rupture) will be recorded in participant files and reported in the final manuscript.

### Data analysis

Statistical analyses will be undertaken using SPSS software (version 14.0 or later, SPSS Corporation, Chicago, IL, USA). Continuous data will be explored for normality using standard tests to satisfy the assumptions of parametric statistics. All analyses will be conducted on an intention-to-treat basis and missing follow-up data will be replaced with baseline observations carried forward (BOCF) (i.e. baseline data will be carried forward in circumstances where follow-up observations are missing). In comparison to a last observation carried forward (LOCF) approach, BOCF has been shown to provide a more conservative estimate when analysing the treatment effect of pain-relief medication [36]. Continuous outcomes with a normal distribution will be analysed using a linear regression technique with baseline measurements adjusted for by the analysis of covariance model (ANCOVA) [37]. If data is found to be not normally distributed, transformation will be attempted. However, if data is still not normally distributed after transformation, non-parametric statistical tests will be used. Other data (nominal or ordinal) will be analysed using appropriate non-parametric statistical tests. Statistical significance for hypothesis tests will be set at the conventional level of 0.05.

## Discussion

### Mode of action

In the treatment of musculoskeletal disorders, corticosteroid injection is typically used to inhibit synthesis of arachidonic acid from membrane phospholipids, thereby suppressing prostaglandin-mediated inflammation and pain [38]. However, as described previously, histological studies indicate that plantar fasciitis is predominantly a degenerative disorder, with limited involvement of chronic inflammatory processes. Consistent with these findings, absence of inflammation is also reported in the tendinopathy literature [14], and the precise mechanism of pain in tendinopathy remains uncertain [39]. Several alternatives to prostaglandin-mediated pain have been suggested in relation to tendon models, including neurovascular in-growth [40], up-regulation of excitatory neurotransmitters (e.g. substance P, glutamate and acetylcholine) [14,39-41], and increased presence of biochemical irritants (e.g. chondroitin sulfate) [39].The action of corticosteroids on these mechanisms is currently unclear [42], however, corticosteroids have been shown to inhibit fibroblast proliferation and expression of ground substance proteins [14,38]. It is possible that these known effects may be of benefit in the treatment of plantar fasciitis, as increased fibroblast proliferation and excessive secretion of proteoglycans are commonly reported features of the condition. Based on similar reasoning, corticosteroids have been suggested as potentially beneficial for treatment of early-stage tendinopathy [14].

### Choice of corticosteroid

Selection of a particular corticosteroid agent for local injection varies across disciplines [43] and geographic regions [44], with limited evidence available to assist in decision-making. In relation to treatment outcomes, systematic reviews of randomised trial data have revealed no difference in clinical efficacy between various corticosteroid types [45,46]. Nonetheless, when selecting a corticosteroid for treatment of soft tissue disorders, guidelines in the rheumatology literature recommend use of an agent with high tissue solubility [42], and avoidance of fluorinated compounds [47]. When corticosteroids are injected locally, duration of action is inversely related to solubility, with high solubility agents (phosphates) having a shorter duration of action, and low solubility agents (acetates) a longer duration of action [42,48]. Accordingly, high solubility corticosteroids (e.g. dexamethasone phosphate) are thought to reduce the risk of post-injection flare and soft -tissue atrophy [49,50].

Fluorination of a corticosteroid molecule improves anti-inflammatory (glucocorticoid) action and decreases sodium retaining (mineralocorticoid) activity, thereby improving the anti-inflammatory potency of the drug while reducing systemic side effects [51]. Despite these advantages, comparisons of fluorinated versus non-fluorinated corticosteroids have demonstrated various effects by which fluorinated agents increase collagen degradation [52-54]. In addition, case-series reports of adverse events following corticosteroid injection, such as rupture of the plantar fascia, have largely involved the fluorinated corticosteroid triamcinolone [55,56]. However, this drug is also the most insoluble (and therefore longest acting) injectable corticosteroid available [48,57], and adverse events following use of shorter-acting fluorinated agents are less frequently reported. With this in mind, it is likely that injection of a corticosteroid that is both fluorinated and relatively insoluble should be avoided when treating soft tissue disorders.

Accordingly, two corticosteroids were considered for use in this trial: (i) methylprednisolone acetate (non-fluorinated, moderate acting) and (ii) dexamethasone sodium phosphate (fluorinated, shorter acting). However, as normal saline solution appears as a clear and colourless liquid, blinding of the investigator performing the injections was considered unachievable with use of an acetate compound. Therefore, dexamethasone sodium phosphate was considered the most appropriate corticosteroid for use in the trial.

Clinician surveys have revealed that combining corticosteroid and local anesthetic solutions prior to soft tissue injection is a widely adopted practice [19,44,58]. Reported benefits of this include provision of temporary pain relief, dilution of potentially harmful corticosteroid crystals (acetates only), and confirmation of accurate solution deposit [59]. Despite this common practice, mixing of corticosteroid solution will not occur in this trial as regional anaesthesia will be performed prior to plantar fascia injections.

### Plantar fascia thickening

Fusiform thickening of the plantar fascia is a well established feature of plantar fasciitis. According to a meta-analysis of diagnostic imaging studies [28], people with plantar heel pain are over 100 times more likely to have an abnormally thickened (> 4.0 mm) plantar fascia compared with asymptomatic controls (odds ratio = 105.11, 95% confidence interval = 3.09 to 3577.28, *P *= 0.01). Abnormal thickening is also reported in the tendinopathy literature, and is thought to be the result of increased secretion of ground substance proteins (e.g. proteoglycans) and subsequent tissue oedema [14]. These changes are considered a response to acute tensile overload and may lead to reduced tissue stress by increasing cross-sectional area [14]. However, it is also noted that increased thickening is not a feature of normal tendon adaptation to chronic loading regimens [14,60], thereby suggesting that changes in tendon thickness are related to abnormal tissue substance.

In response to corticosteroid injection, plantar fascia thickness values have been shown to decrease significantly as early as two weeks [24] and one month [61] following treatment. One of these studies also reported a correlation between decreased plantar fascia thickness and pain relief (Pearson *r *= 0.61, *P *< 0.001) [61]. However, as neither of these studies made comparisons to a control group, it is possible that the findings were partly due to the condition's natural course. Nonetheless, a recent randomised trial reported a significant decrease in pain and plantar fascia thickness following injection with botulinum toxin, when compared to placebo injection [62]. This evidence suggests that plantar fascia thickness measurements can provide useful objective data, and may assist in identifying overall improvement in the condition.

### Posterior tibial nerve block

Studies investigating the clinical benefit of regional anaesthesia given prior to plantar fascia injection have produced inconsistent findings. Two trials report no difference in overall procedure comfort between plantar fascia injections given after tibial nerve blockade, and plantar fascia injections given alone [22,63]. Explanations for these counterintuitive findings include: (i) pain and paraesthesia commonly occur during a nerve block from contact between the needle tip and nerve fascicle [22,64] and (ii) successful anaesthesia of the posterior tibial nerve is difficult to achieve, especially within a short period of time [22,65]. In contrast, one study found that a posterior tibial nerve block effectively reduces pain during the plantar fascia injection itself [66]. Furthermore, in comparison to a landmark-based technique, use of ultrasound guidance during regional anaesthesia has been shown to reduce the occurrence of paraesthesia and inadvertent intravascular injection, while improving block onset time and success rates [64,67,68]. With this in mind, we have chosen to perform an ultrasound-guided posterior tibial nerve block prior to plantar fascia injection.

### Inclusion of stretching program

Combination of placebo treatment and double-blinding will be undertaken to control for intervention effects not due to the pharmacological action of corticosteroids. Prescription of a standardised stretching program has been introduced in order to compensate for the presence of a placebo group, thereby ensuring every participant receives treatment for their condition.

## Conclusion

This will be a randomised trial investigating the effect of dexamethasone injection on pre-specified treatment outcomes in people with plantar fasciitis. It is possible that some aspects of the protocol will limit the extent to which findings can be generalised to routine clinical settings. Features most likely to limit external validity include provision of regional anaesthesia, use of an ultrasound-guided injection technique, and injection of plain corticosteroid solution (without mixing). Nonetheless, this trial will provide high quality evidence for the pharmacological effect of corticosteroids in the treatment of plantar fasciitis. Furthermore, within the parameters of this protocol, the trial findings will be used to make evidence-based recommendations regarding the use of corticosteroid injection for treatment of this condition.

## Competing interests

HBM and KBL are Editor-in-Chief and Deputy Editor-in-Chief, respectively, of the *Journal of Foot and Ankle Research*. It is journal policy that editors are removed from the peer review and editorial decision making processes for papers they have co-authored.

## Authors' contributions

AMM and KBL conceived the study idea and designed the trial protocol. AMM obtained funding for the study, drafted the protocol manuscript and is the chief investigator. KBL obtained funding for the study and commented on the draft manuscript. HBM, MFG, ARB and ADM assisted in designing the trial protocol and commented on the draft manuscript. All authors read and approved the final manuscript prior to submission.
